# Evolving migraine management: Lithuania's telemedicine experience

**DOI:** 10.3389/fneur.2024.1388100

**Published:** 2024-05-22

**Authors:** Saulius Andruskevicius, David Petrosian, Austeja Dapkute, Mantas Jokubaitis, Kristina Ryliskiene

**Affiliations:** ^1^Center of Neurology, Vilnius University, Vilnius, Lithuania; ^2^Faculty of Medicine, Vilnius University, Vilnius, Lithuania

**Keywords:** migraine, headache, telehealth, telemedicine, remote consultations

## Abstract

**Introduction:**

COVID-19 challenges have underscored the potential of telemedicine in migraine management. This study focuses on assessing patients' telemedicine experience for headache management in Lithuania and identifying key barriers and facilitators for its wider use.

**Methods:**

A nationwide e-survey was conducted in 2023 via the Lithuanian Association of Migraine Patients' website, social media platforms, websites of public and private healthcare facilities, and migraine self-help groups. The survey covered sociodemographics, migraine characteristics, previous experience with teleconsultations for headaches with neurologists and general practitioners (GP), perceived advantages and disadvantages of telehealth, and preferred future consultation types.

**Results:**

Eight hundred and forty seven respondents with a confirmed migraine diagnosis were analyzed. The majority were female (97.2%), with a median age of 35 (IQR 30–42) years and an average of 5 (IQR 3–9) monthly headache days (MHDs). 7.0% of respondents had chronic migraine (CM). Prior teleconsultations for headaches were reported by 35.2% of respondents, 26.2% with a GP and 17.0% with a neurologist (*p* < 0.0001). Teleconsultation outcomes included continuation of a prescribed treatment (84.7% for GPs and 83.3% for neurologists, *p* = 0.7295), initiation of new acute medications (12.2% for GPs with 70.4% reported as effective and 27.1% for neurologists with 84.6% effective, *p* = 0.0005 and *p* < 0.0001, respectively). Reasons for not undergoing remote neurology consultations: the lack of inquiry (69.7%), unavailability from neurologists (18.1%) and respondent's opposition to remote consultations (12.2%). Patients evaluated their experience with remote neurology services better than that of GPs (*p* = 0.0289). 67.3% of respondents preferred a mixed-mode approach for future consultations. In-person-only preference (29.0%) correlated with multiple factors, including history of remote primary neurology consultations (OR 5.89, *p* = 0.0022), lower education (OR 2.20, *p* = 0.0001), physically demanding work (OR 1.95, *p* = 0.0001), and number of drawbacks in telemedicine identified (OR 1.30, *p* < 0.0001), and worse experience of a prior remote GP consultation (OR 0.704, *p* < 0.0001). The main indicator of preference for remote-only consultations was the perception of fewer telemedicine disadvantages (OR 0.503, *p* = 0.0007).

**Conclusions:**

Our findings confirm that telemedicine contributes to effective migraine management and is used limitedly in Lithuania. Despite one-third of respondents having experienced teleconsultations, significant barriers remain. Our study highlights a clear preference for a hybrid consultation type.

## 1 Introduction

Migraine is a primary headache disorder with complex pathophysiology and variable clinical manifestations. It impacts approximately 15% of population worldwide and up to 35% in Europe annually (1, 2). Migraine ranks as the world's second leading cause of disability and the primary cause among young women (3). Considering its widespread and growing prevalence leading to substantial burden, migraine management necessitates specialized healthcare services, which vary in accessibility and cost-effectiveness across and within countries (4, 5). Accessibility to migraine care is hindered by geographical constraints, a scarce number of headache specialists, and the requirement for frequent patient follow-ups (6–9). These barriers were exacerbated by the COVID-19 pandemic, which severely restricted traditional in-person medical interactions, underscoring the critical need for alternative healthcare delivery methods (10). In response, telemedicine has emerged as a viable solution, presenting the opportunity to overcome these challenges by enabling remote healthcare services and ensuring continuity and accessibility in migraine care (11, 12). With ongoing technological advancements, telemedicine use is expected to increase, particularly in headache management, where its implementation was already notable even before the pandemic (11, 13, 14).

Previous studies suggest that telemedicine for headache management is non-inferior to traditional in-person consultations in effectiveness. Additionally, the advantages of remote healthcare services, particularly saved time and expenses, are especially significant for patients with limited access to healthcare (15). To evaluate the impact of telemedicine on migraine management, we conducted a national e-survey in Lithuania. We hypothesized that telemedicine yields satisfactory disease management results for migraine patients and is underutilized in Lithuania. The study aimed to assess migraineurs' previous experiences with telemedicine and to identify potential barriers and facilitators for its wider implementation in the future.

## 2 Materials and methods

### 2.1 Survey structure

An online survey was designed specifically for this study and administered via Google Forms platform. The first part focused on patient sociodemographic information, including age, sex, marital and parental status, employment status, type of work, education, digital literacy from 0 (no skills) to 10 (expert user), distance from home to a neurologist, and general practitioner (GP), preferred transportation methods to healthcare centers, private health insurance status, and the use of medication for concomitant conditions. In the second part of the survey, we collected migraine-related data, including the age of onset, type of migraine, average pain intensity on a scale from 1 (mild discomfort) to 10 (severe pain) when acute migraine treatment is not effective, number of monthly headaches days (MHDs), typical acute and preventive medications used, monthly days with acute headache medications. The survey also included questions about previous experiences with remote consultations for headaches, registration and communication types, factors influencing respondents' choice of telehealth and difficulties encountered, consultation outcomes (diagnosis of a new headache disorder, treatment adjustments, referrals to the emergency department, and sick-leave prescriptions), an overall level of satisfaction and generally perceived advantages and disadvantages of telehealth. Patients were asked to choose their preferred future consultation type from “online-only,” “in-person-only,” and “mixed-type.” The questionnaire featured options for structured responses and included a provision for respondents to add their own text entries to elaborate or introduce alternative choices.

### 2.2 Study design

**Figure 1:**
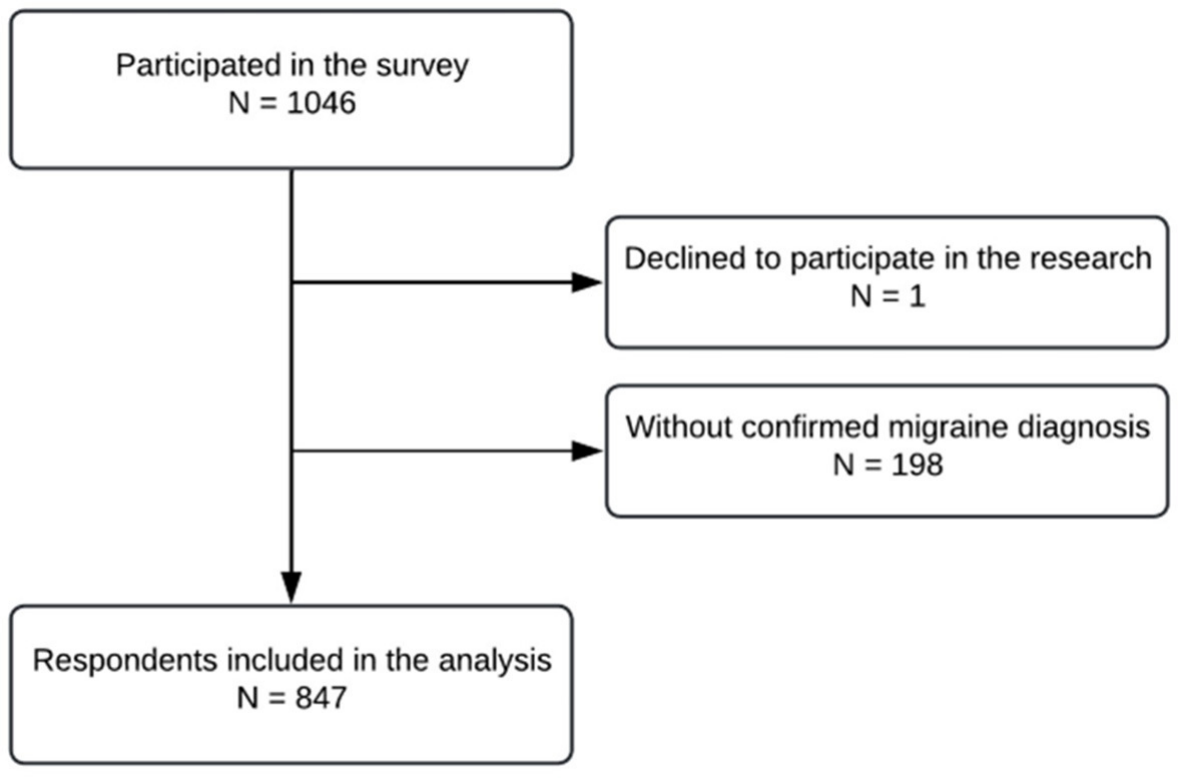
Flowchart of participants in the survey.

An anonymous nationwide e-survey was distributed via the Lithuanian Association of Migraine Patients' website, social media platforms, official websites of healthcare facilities, and online migraine self-help groups. The study was conducted between January and February 2023. Participants were informed about the purpose of the study and their consent was obtained as an integral part of the survey. A total of 1046 individuals responded to the inquiry, and 847 patients with confirmed migraine diagnosis were selected for further analysis (Figure 1). As the anonymized nature of the data collection prevented individual identification, obtaining ethics approval was not required in accordance to The Vilnius Regional Biomedical Research Ethics Committee, adhering to Principle 26 of the General Data Protection Regulation.

### 2.3 Statistical analysis

In this study, we employed bivariate and multivariate analysis to investigate relationships within the collected data. As tested with Shapiro-Wilk test for sample distribution normality, none of our variables followed normal distribution, thus all continuous data are presented using median and interquartile range (IQR). The Mann Whitney U test was applied for comparisons between two continuous variables. For more than two variables, The Kruskall-Wallis test was used. Categorical variables were compared using the Chi-square test, with an exception for variables with sample size of <20 per group, where the Fisher's exact test was applied. The Wald test was used for assessing the significance of regression coefficients. A *p*-value threshold for statistical significance was set to 0.05. Future appointment selection predictors were controlled for the false discovery rate (FDR), reducing the significance threshold to *p* < 0.003. All statistical analyses were performed using R 4.3.2 and SPSS 27.0.

## 3 Results

### 3.1 Sociodemographic data

Of the 847 analyzed responders, 823 were female (97.2%), with an average age of 35 (IQR 30–42) years. Sociodemographic data of our study sample are presented in Table 1. Two hundred and nighty eight (35.2%) patients had a diagnosed migraine with aura, 59 individuals (7.0%) had a diagnosis of chronic migraine (CM). The median of monthly headache days (MHDs) was 5 (IQR 3–9). The summary of migraine-associated characteristics is presented in Table 2.

**Table 1:** Sociodemographic data of the study participants.

**Variable**	**Overall (*n =* 847)**	**Previously consulted for headaches remotely (*n =* 298)**	**Not consulted for headaches remotely (*n =* 549)**	***P* value**
Female, *n* (%)	823 (97.2%)	292 (98.0%)	531 (96.7%)	0.3993
Age (median (IQR), years)	35 (30–42)	35 (30–42)	35 (31–42)	0.3785
Raising <18 y.o. children, *n* (%)	472 (55.7%)	164 (55.0%)	308 (56.1%)	0.8208
Living with a partner, *n* (%)	710 (83.8%)	252 (84.6%)	458 (83.4%)	0.7396
Digital literacy from 1 (no skills) to 10 (expert user) (median (IQR)	9 (8–10)	9 (8–10)	9 (8–10)	0.9322
Private health insurance, *n* (%)	332 (39.2%)	111 (37.2%)	221 (40.3%)	0.4341
Distance to a GP [median (IQR), km]	4 (2–8)	5 (2–10)	4 (2–9)	0.3186
Distance to a neurologist [median (IQR), km]	6 (3–15)	7 (3–20)	6 (3–12)	0.1311
**Education**				
Higher, *n* (%)	715 (84.4%)	250 (83.9%)	465 (84.7%)	0.8337
Secondary/vocational, *n* (%)	126 (14.9%)	45 (15.1%)	81 (14.8%)	0.9727
Unfinished secondary, *n* (%)	6 (0.7%)	3 (1.0%)	3 (0.5%)	0.7386
**Employment**				
Mentally demanding job, *n* (%)	702 (82.9%)	243 (81.5%)	459 (83.6%)	0.5056
Physically demanding job, *n* (%)	189 (22.3%)	74 (24.8%)	115 (20.9%)	0.2261
Remote job, *n* (%)	205 (24.2%)	60 (20.1%)	145 (26.4%)	0.0508
On-site job, *n* (%)	642 (75.8%)	238 (79.9%)	404 (73.6%)	0.0508
Student, *n* (%)	64 (7.6%)	30 (10.1%)	34 (6.2%)	0.0573
Retired, *n* (%)	8 (0.9%)	2 (0.7%)	6 (1.1%)	0.8149
Unemployed, *n* (%)	41 (4.8%)	15 (5.0%)	26 (4.7%)	0.9799
**Mode of transport to reach healthcare facility**				
Private car, *n* (%)	633 (74.7%)	229 (76.8%)	404 (73.6%)	0.3376
Public transport, *n* (%)	114 (13.5%)	34 (11.4%)	80 (14.6%)	0.2370
Walking, *n* (%)	86 (10.2%)	33 (11.1%)	53 (9.7%)	0.5932
Taxi, *n* (%)	13 (1.5%)	3 (1.0%)	10 (1.8%)	0.5297
Others, *n* (%)	3 (0.4%)	0 (0.0%)	3 (0.5%)	0.5011

n, number; IQR, interquartile range; y.o., years old; GP, general practitioner; km, kilometers.

**Table 2:** Migraine characteristics and their comparison between patients with and without previous telemedicine experience.

**Variable**	**Overall (*n =* 847)**	**Previously consulted for headaches remotely (*n =* 298)**	**Not consulted for headaches remotely (*n =* 549)**	***P* value**
Migraine history [median (IQR), years]	18 (14–25)	15 (8–22)	17 (10–24)	0.1261
Migraine without aura, *n* (%)	549 (64.8%)	189 (63.4%)	360 (65.6%)	0.5313
Migraine with aura, *n* (%)	298 (35.2%)	109 (36.6%)	189 (34.4%)	0.5313
Chronic migraine, *n* (%)	59 (7.0%)	33 (11.1%)	26 (4.7%)	**0.0005**
Monthly headache days [median (IQR)]	5 (3–9)	6 (4–10)	5 (3–8)	**<0.0001**
Monthly days of acute migraine medication use [median (IQR)]	5 (3–9)	6 (3–10)	5 (3–8)	**<0.0001**
Use of acute migraine medications, *n* (%)	816 (96.3%)	291 (97.7%)	525 (95.6%)	0.1792
Use of triptans, *n* (%)	555 (65.5%)	229 (76.8%)	326 (59.4%)	**<0.0001**
Use of oral NSAIDs, *n* (%)	547 (64.6%)	192 (64.4%)	355 (64.7%)	0.9459
Use of intramuscular NSAIDs, *n* (%)	297 (35.1%)	127 (42.6%)	170 (31.0%)	**0.0007**
Pain level when analgesics are ineffective (1–10 scale) [median (IQR)]	8 (7–9)	8 (7–9)	8 (7–9)	**0.0025** ^*^
Use of preventive medications, *n* (%)	221 (26.1%)	109 (36.6%)	112 (20.4%)	**<0.0001**
Monoclonal anti-CGRP/Rc antibodies, *n* (%)	139 (16.4%)	74 (24.8%)	65 (11.8%)	**<0.0001**
Beta-blockers, *n* (%)	81 (9.6%)	35 (11.7%)	46 (8.4%)	0.1117
Antidepressants, *n* (%)	61 (7.2%)	33 (11.1%)	28 (5.1%)	**0.0013**
Antiepileptic drugs, *n* (%)	14 (1.7%)	7 (2.3%)	7 (1.3%)	0.2658
Regular use of medications for comorbidities, *n* (%)	289 (34.1%)	117 (39.3%)	172 (31.3%)	**0.0201**
Cardiovascular disorders, *n* (%)	126 (14.9%)	46 (15.4%)	80 (14.6%)	0.7357
Gastrointestinal disorders, *n* (%)	120 (14.2%)	45 (15.1%)	75 (13.7%)	0.5662
Other neurologic or psychiatric disorders, *n* (%)	110 (13.0%)	53 (17.8%)	57 (10.4%)	**0.0022**

^*^P-value reflects distribution differences, with means of 8.02 (consulted) vs. 7.65 (not consulted). n, number; IQR, interquartile range; NSAIDs, non steroidal anti-inflammatory drugs; CGRP, calcitonin gene-related peptide; Rc, receptor. Bold values indicate statistically significant, *p* < 0.05.

### 3.2 Previous experience with telemedicine for migraine care

A total of 298 respondents (35.2%) were consulted by a healthcare professional remotely for migraines. Among them, 222 patients were consulted by GPs, 144 were consulted by neurologists (26.2% and 17.0% of all respondents, respectively, *p* < 0.0001). A higher previous exposure to telemedicine for headaches positively correlated with more MHDs [6 (1–10) vs. 5 (3–8), *p* < 0.0001], higher use of triptans (76.8% vs. 59.4%, *p* < 0.0001), monoclonal antibodies against calcitonin gene related peptide or receptor (anti-CGRP/Rc) (24.8% vs. 11.8%, *p* < 0.0001) and antidepressants (11.1% vs. 5.1%, *p* = 0.0013) for migraine prevention. Consultation rate was also higher for chronic migraineurs (*n* = 33, 11.1% vs. *n* = 26, 4.7%, *p* = 0.0005). The comparison of respondent characteristics with and without prior experience of telemedicine is summarized in Table 2.

A preference for engaging in remote consultations with neurologists was demonstrated by individuals who had longer migraine history (17 (15–25.25) years vs. 15 (8–22) years, *p* = 0.0061) and using preventative migraine medications (42.1% vs. 26.6%, *p* = 0.0180), including anti-CGRP/Rc therapies (32.9% vs. 14.9%, *p* = 0.0017). Patients regularly using medications for comorbidities consulted GPs more often than neurologists (37.7% vs. 36.8%, *p* < 0.0001). During remote consultations, neurologists' prescribed new treatments were found to be more effective in comparison to GPs' (84.6% vs. 70.4%, *p* < 0.0001). Compared to neurologists, GPs more frequently referred patients to emergency departments (7.7% vs. 4.9%, *p* < 0.001) and prescribed sick leaves (28.4% vs. 6.3%, *p* < 0.001). Table 3 summarizes the data reported by respondents regarding previous remote consultations for headaches and the resulting outcomes.

**Table 3:** Respondent-reported data on previous remote consultations for headaches and their outcomes.

**Variable**	**GPs**	**Neurologists**	***P* value**
Number of consulted patients (*n*, %)	222 (26.2%)	144 (17.0%)	**<0.0001**
Primary consultation (*n*, %)	78 (35.2%)	27 (18.8%)	**0.0011**
Number of consultations [median (IQR)]	2 (1–5)	2 (1, 2)	0.2083
Had an opportunity to choose a method (*n*, %)	38 (17.1%)	34 (23.6%)	0.1268
Would have preferred a video call (*n*, %)	74 (33.3%)	56 (38.9%)	0.278
Time from registration to consultation [median (IQR), days]	5 (2–10)	14 (5–30)	**<0.0001**
Consultation duration [median (IQR), minutes]	10 (5–10)	10 (5–15)	**0.0002**
**Registration method**			
Telephone (*n*, %)	175 (78.8%)	85 (38.3%)	**<0.0001**
Online registration system (*n*, %)	64 (28.8%)	33 (22.9%)	0.2106
On-site reception (*n*, %)	9 (4.1%)	25 (17.4%)	**<0.0001**
**Consultation method**			
Telephone (*n*, %)	216 (97.3%)	134 (93.1%)	0.067
Video call (*n*, %)	1 (0.5%)	7 (4.9%)	**0.0071**
Email (*n*, %)	–	2 (1.4%)	–
**Consultation price**			
Free of charge (*n*, %)	214 (96.4%)	123 (85.4%)	
Lower than in-person (*n*, %)	6 (2.7%)	11 (7.6%)	**0.0004**
The same as in-person (*n*, %)	2 (0.9%)	10 (6.9%)	
**Consultation outcomes**			
Continuation of previously prescribed treatment (*n*, %)	188 (84.7%)	120 (83.3%)	0.7295
Initiation of new acute medications (*n*, %)	27 (12.2%)	39 (27.1%)	**0.0005**
Medications were effective (*n*, % of prescribed acute treatment)	19 (70.4%)	33 (84.6%)	**<0.0001**
Initiation of new preventive treatment (*n*, %)	–	40 (27.8%)	–
Medications were effective (*n*, % of prescribed preventive treatment)	–	28 (70.0%)	–
Prescription of sick leave (*n*, %)	63 (28.4%)	9 (6.3%)	**<0.0001**
Referral to an emergency department (*n*, %)	17 (7.7%)	7 (4.9%)	0.3882
General evaluation (1–10 scale) [median (IQR)]	8 (7–10)	9 (8–10)	**0.0289**

n, number; IQR, interquartile range; GP, general practitioner. Bold values indicate statistically significant, *p* < 0.05.

### 3.3 Barriers and facilitators

**Figure 2:**
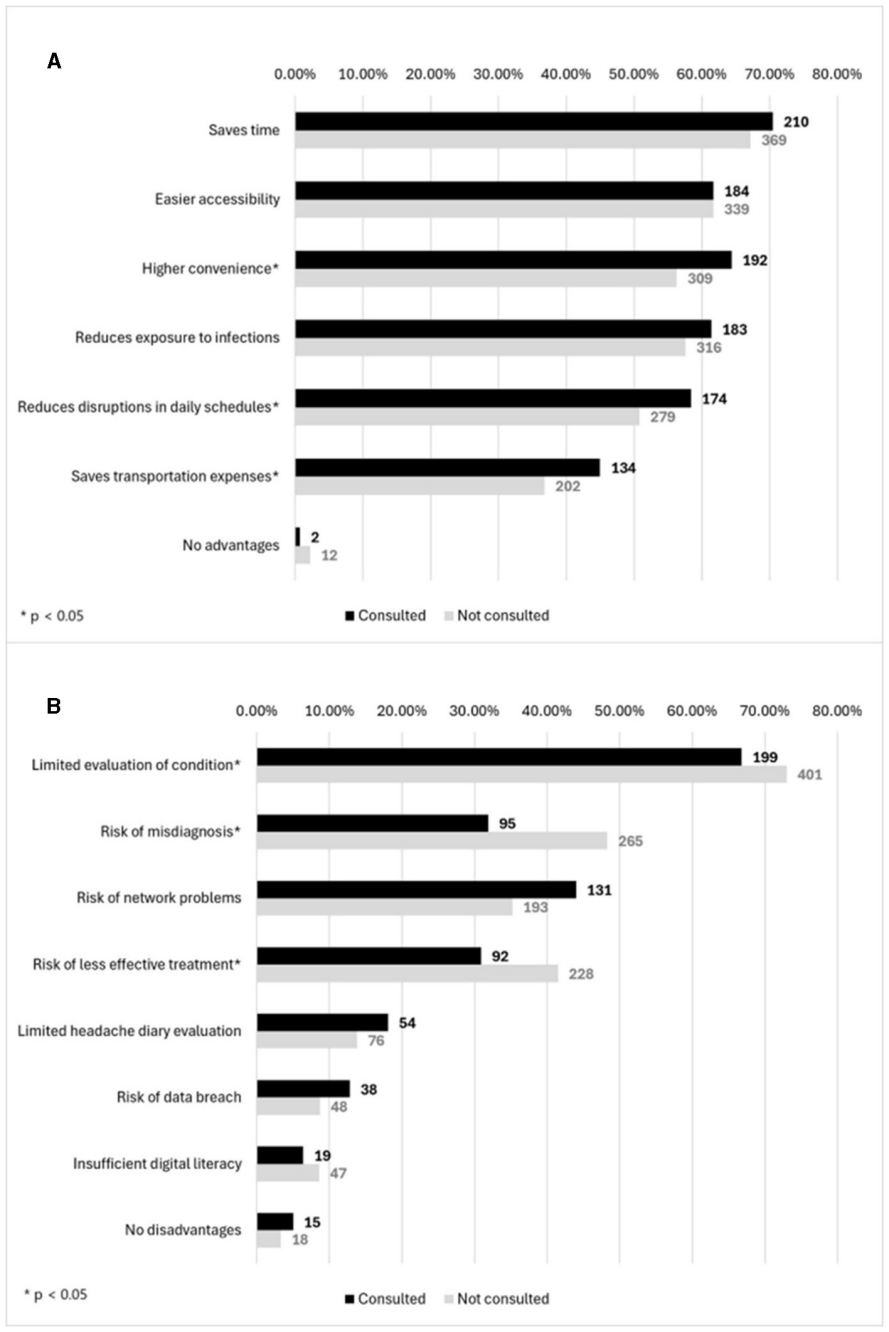
**(A)** Distribution of advantages and **(B)** disadvantages of patients who engaged and not engaged in telemedicine.

Challenges encountered by respondents regarding remote GP consultations included the difficulty registering by phone (*n* = 90, 40.5%), lack of available appointment times (*n* = 84, 37.8%) and non-user-friendly online registration system (*n* = 29, 13.1%). 38.3% (*n* = 85) reported no issues. Considering neurology consultations, reported issues consist of the lack of available appointment times (*n* = 52, 15.1%), difficulty registering by phone (*n* = 44, 12.8%) and a non-user-friendly online registration system (*n* = 23, 6.7%). 20.3% (*n* = 70) of those who had experienced remote neurology consultations reported no issues. The most prominent reasons for not undergoing remote neurology consultations were not inquiring about the possibility of telemedicine services (*n* = 490, 69.7%), neurologists not providing remote consultations (*n* = 127, 18.1%), and respondent's personal stance against remote consultations (*n* = 86, 12.2%). Perceived advantages and disadvantages of remote services for headache management were identified and presented in Figure 2. Generally, patients who had previous experience with telemedicine noted a higher number of advantages in telemedicine [4 (2–5) for consulted and 3 (1–5) for non-consulted, *p* = 0.0273].

### 3.4 Future preferences

67.3% (*n* = 570) of participants indicated a preference for a mixed-mode headache consultations in the future. 29.0% (*n* = 246) of respondents would choose in-person consultations only. Only 31 (3.7%) respondents indicated a preference for telemedicine-only consultations for headache management in the future. The correlations of respondents' sociodemographic and migraine characteristics with the choice of future consultation type are summarized in Table 4.

**Table 4:** Factors impacting the choice of future consultation types.

**Correlating factors**	**Bivariate analysis (Fisher's exact/Kruskal-Wallis tests)**	**Univariate Multinomial Logistic Regression for the preference for in-person-only consultation type (** ***n*** **, %)**				**Univariate Multinomial Logistic Regression for the preference for remote-only consultation type (** ***n*** **, %)**			
	***P*** **-value**	**OR**	**95% CI Lower**	**95% CI Upper**	***P*** **-value**	**OR**	**95% CI Lower**	**95% CI Upper**	***P*** **-value**
Receiving primary neurology consultations remotely	0.0073^†^	5.89	1.89	18.30	**0.0022**	5.53	0.483	63.2	0.1690
Level of education^a^	**0.0003** ^†^	2.20	1.47	3.27	**0.0001**	0.001	3.12 × 10^−68^	1.29 × 10^61^	0.9220
Physically demanding work	**0.0003** ^†^	1.95	1.39	2.74	**0.0001**	0.634	0.217	1.85	0.4040
Number of disadvantages in telemedicine identified	**<0.0001** ^‡^	1.30	1.18	1.44	**<0.0001**	0.503	0.337	0.748	**0.0007**
Higher number of medication-use days per month	0.0031^‡^	1.05	1.02	1.08	**0.0007**	1.03	0.965	1.10	0.3710
Higher evaluation of a previous remote experience with a GP (scale 1-10)	**<0.0001** ^‡^	0.704	0.609	0.814	**<0.0001**	1.18	0.767	1.81	0.4530
Remote work	**0.0011** ^†^	0.58	0.394	0.844	0.0046	2.02	0.967	4.23	0.0612

FDR-corrected significance level *p* < 0.003.

^a^Unfinished secondary education/secondary or vocational school/higher education;^†^Fisher's exact test for categorical variables;^‡^Kruskal-Wallis test for numerical variables; n, number; OR, odds ratio; CI, confidence interval; GP, general practitioner. Bold values indicate statistically significant, *p* < 0.003.

## 4 Discussion

Consistent with our hypothesis, the results of this study highlight a relatively low engagement in telemedicine for migraine management in Lithuania. Among those surveyed, 35.2% had at least one remote consultation, yet only 17% were consulted by a neurologist. This finding indicates a significant gap in telehealth usage in Lithuania, compared with 57.5% of respondents participating in telemedicine services reported in a survey by the American Migraine Foundation in 2020 (16). The modest adoption of telemedicine services in Lithuania may be attributed to the country's compact geography, which facilitates easier access to healthcare facilities. In contrast, larger nations, such as Norway and the USA, where healthcare facilities can be more remote, rely more heavily on telemedicine (8, 17, 18).

This study demonstrated that patients with more MHDs, chronic migraine and preventive treatment use, also with more severe headaches influenced by higher pain scores and increased monthly acute medication use were more frequently consulted remotely by GPs and neurologists. Similar outcomes, underscoring the association between migraine severity and the incidence of consultations with healthcare professionals, have been corroborated by other studies (19, 20). Moreover, our findings, which reveal that GPs consult headache patients remotely more frequently than neurologists, partially align with the migraine care model proposed by Ashina et al., which advocates for the management of the majority of headache patients within primary care settings (1). However, the implementation of these recommendations in Lithuania is facing significant obstacles. Our healthcare system mandates neurologists as the initial prescribers of reimbursed triptans for migraine attack treatment. Additionally, anti-CGRP/Rc monoclonal antibodies, a leading preventative treatment among those surveyed, are reimbursed for both episodic and chronic migraine prevention, necessitating regular neurologist follow-up every 3–9 months (1, 7). Therefore, our findings indicate a lower frequency of consultations with neurologists than might be anticipated or necessary for optimal care, pointing to a potential shortfall in migraine management within the Lithuanian healthcare framework. The study's results suggest several key factors contributing to the underutilization of teleneurology services. A significant portion of our study participants without telemedicine experience reported disinterest or even a personal opposition against remote consultations (69.7% and 12.2%, respectively) and 18% noted a scarcity of available services. These findings are contrary to other studies demonstrating significantly higher acceptance rates of telehealth in headache care from both patients and healthcare providers (16–18). The reluctance to adopt telemedicine in specific patient cohorts remain, mainly influenced by the lack of technological skills, data safety concerns, anticipated negative outcomes and other (21).

Multiple studies have supported the effectiveness and patient satisfaction with teleconsultations, in addition to some indicating no significant difference from in-person visits (11, 15, 22–24). In our study, the rates of prescribing new treatments proved to be lower compared to those reported in similar study by Chiang et al. (16). However, our data indicate that the overall experience with remote neurology consultations was more favorable, as neurologist prescriptions were 84.6% effective for acute and 70.0% for preventative medications, compared to 70.4% of effective GP-prescribed acute treatment. This discrepancy underscores the potential for improvement in GP training on headache management or in refining patient triage for specialist care. Other studies have reported significant treatment success through telemedicine, with effectiveness rates ranging from 40% to over 50% in Norway and China (22, 25). The marked diverge of our results from these findings could be explained by the difference in efficacy measures, notably, in our study, only perceived efficacy was evaluated in contrast to quantitative efficacy measures in previous studies. The observed higher referral rates to emergency departments (EDs) by GPs compared to neurologists may reflect GPs' roles as a primary point of contact for headache patients, possibly encountering more acute cases or exercising greater caution. This finding supports the expansion of telemedicine services to reduce headache-related ED visits (16). Moreover, there is a clear need for precise and universally applicable guidelines for assessing neurological emergencies and for strengthening communication between neurologists and GPs, an issue that could be addressed by adopting robust guidelines recommended in the Consensus Statement by Eigenbrodt et al. (7).

The necessity for some form of physical evaluation in headaches remains, even in a remote setting. Robblee et al. have significantly contributed to this aspect by detailing a process for remote physical examination, thereby enhancing the effectiveness of teleneurology in the management of primary headache disorders (26). Our study shows that 81.2% of remote neurologist consultations focused on follow-up care, mainly for treatment prescriptions. This indicates that initial visits are typically held in person, often necessitating physical examinations. Such a pattern may explain why some physicians prefer telephone follow-ups to primary remote consultations (17, 27). Nonetheless, the reliance on teleconsultations has its limitations as they cannot entirely replace in-person appointments, especially for crucial procedures like fundus assessment, which cannot be performed remotely (13, 26).

Respondents with previous teleconsultation experience were more likely to acknowledge the benefits of remote consultations, such as reduced transportation costs, the convenience of receiving consultations from home, and minimal disruption to daily schedules. Similar advantages, including improved access to headache specialist, a favorable safety profile and the option of an in-person follow-up, were also described in other publications (13, 28–31). Additionally, some evidence from study in Switzerland suggest that positive experiences with telemedicine can accelerate its broader implementation (32). Conversely, our data demonstrate that participants without telemedicine experience expressed greater concerns, including inadequate assessments, higher risk of misdiagnoses, and unsuitable treatments. Other perceived disadvantages previously reported in the literature include the absence of direct interaction, technical difficulties and costs associated with technology use (13, 28–32).

Our results align with previous studies (28, 30, 33) with 67.3% of respondents expressing a preference for mixed-mode appointments in the future. Contrary to the United States, where only 7.1% of headache patients declined telemedicine (16), 29.0% of our respondents expressed a preference for exclusively in-person consultations in the future. Manual work, lower education levels, higher medication intake were identified as key correlates that have also been summarized in other reviews (21, 34). An intriguing finding from our study emerged suggesting that respondents who had their primary neurology appointments remotely were less likely to choose teleconsultations in the future. A plausible explanation for this could be the absence of physical interactions, a component that patients often deem essential, underscoring the advantage of a hybrid approach that combines initial in-person consultations (13, 29, 30, 33). Furthermore, our study revealed that patients who identified more disadvantages in telemedicine care and had lower evaluations of previous remote experiences with a general practitioner (GP) showed a greater preference for in-person visits. This supports the findings of Reinhardt et al. in a scoping review, indicating that negative remote experiences influence future preferences (21). Notably, the potential impact of specific medical procedures, such as onabotulinumtoxinA injections and peripheral nerve blocks, on these preferences was also considered in our investigation. However, given their very rare application in Lithuania, it is unlikely that these treatments have significant effect on the inclination toward hybrid or contact-only consultations as observed among our respondents.

A mere 3.7% of participants indicated a preference for telemedicine-only consultations, pointing to limited prospects of fully remote headache management. This data diverge from findings in Portugal, where 12% of headache patients selected teleconsultations as their preferred medical visit model (30). In our study, the sole significant indicator suggesting a heightened patient preference for remote-only consultations was the perception of fewer disadvantages associated with telemedicine.

The strengths of this study are highlighted by its geographically comprehensive participant base from all Lithuanian regions and a substantial sample size of 847 migraineurs. It introduces novel data on the role of telemedicine in headache care, capturing patient experiences and perspectives on the benefits and drawbacks of remote management. These insights are pivotal for policymakers, offering a foundation to tailor healthcare policies toward effective telehealth integration in headache care. This study has some limitations. Internet-based surveys frequently encounter challenges related to demographic representativeness, exacerbated by disparities in age, internet accessibility and digital literacy (35). The lack of interpersonal engagement can contribute to discrepancies in question interpretation. The dependence on self-reported diagnoses and absent external verification introduces a potential bias that casts doubt on the veracity of the migraine diagnosis. There is an absence of detailed data to ascertain whether the choice of consultation method offered to patients applied to the first or follow-up consultations. Furthermore, our investigation does not differentiate between pre-pandemic, pandemic, and post-pandemic periods, marking a limitation that could have offered deeper insights into the evolving dynamics of telemedicine usage. Additionally, the recruitment strategy, leveraging specific online platforms and groups, might have biased the sample toward those more actively engaged in managing their condition.

In conclusion, our findings suggest that telemedicine contributes to effective and satisfactory migraine management, despite its limited use in Lithuania. Individuals who have utilized telemedicine for headache demonstrated a more positive perception toward this technology, recognizing a greater number of advantages, and fewer concerns about misdiagnosis or inappropriate treatment, compared to those who have not been consulted remotely. About one-third of participants remained hesitant about adopting telemedicine in the future, underscoring its potential disadvantages. These findings reinforce the idea that the attitudes and experiences of patients significantly shape their future consultation preferences.

## Data availability statement

The raw data supporting the conclusions of this article will be made available by the authors, without undue reservation.

## Ethics statement

Ethical approval was not required for the studies involving humans because as the anonymized nature of the data collection prevented individual identification, obtaining ethics approval was not required in accordance to the Vilnius Regional Biomedical Research Ethics Committee, adhering to Principle 26 of the General Data Protection Regulation. The studies were conducted in accordance with the local legislation and institutional requirements. Written informed consent for participation was not required from the participants or the participants' legal guardians/next of kin in accordance with the national legislation and institutional requirements. The research was conducted via anonymized internet surveys due to the minimal risk and the preservation of participant anonymity, which inherently protects their privacy and confidentiality.

## Author contributions

SA: Conceptualization, Methodology, Writing – original draft, Writing – review & editing. DP: Conceptualization, Formal analysis, Visualization, Writing – original draft, Writing – review & editing. AD: Formal analysis, Visualization, Writing – original draft, Writing – review & editing. MJ: Writing – original draft, Writing – review & editing. KR: Conceptualization, Methodology, Supervision, Writing – original draft, Writing – review & editing.
